# Special Issue: Metabolic Bone Diseases: Molecular Biology, Pathophysiology and Therapy

**DOI:** 10.3390/ijms24109065

**Published:** 2023-05-22

**Authors:** Anastasia Xourafa, Agostino Gaudio

**Affiliations:** 1 UO di Medicina Interna, Policlinico “G. Rodolico”, Via S. Sofia 78, 95123 Catania, Italy; axourafa@gmail.com; 2 Department of Clinical and Experimental Medicine, University of Catania, 95123 Catania, Italy

Bone is a vital tissue as it carries out various metabolic functions: support of the body, protection of the internal organs, mineral deposit and hematopoietic functions. It has recently been observed that bone tissue also has endocrine functions, producing substances with hormonal activity such as osteocalcin and Fibroblast Growth Factor-23 (FGF-23) [1,2].

As is well known, bone tissue is continuously renewed through the bone remodelling process. The first phase involves bone resorption, mediated by osteoclasts, and the subsequent phase bone formation, mediated by osteoblasts. The continuous succession of these two phases keeps the skeleton young and allows the repair of microcracks [1].

Bone metabolism, which is finely regulated by different biologic signals [1,3], can be affected by various disorders. Metabolic bone diseases include common clinical conditions such as osteoporosis, osteomalacia, and primary hyperparathyroidism, as well as rare conditions such as Paget’s disease, fibrous dysplasia, tumour-induced osteomalacia, osteogenesis imperfecta, and several sclerosing bone diseases [4,5,6].

The identification of mechanisms underlying the pathophysiology of metabolic bone diseases continues to be an area of significant research efforts, attracting scientists from different fields.

For this Special Issue, we received different scientific contributions, spanning in vitro studies to clinical research articles and reviews of the scientific literature.

The paper by Jansen et al., entitled “Increased bone resorption during lactation in pycnodysostosis” [7], explores the alteration of bone metabolism in patients affected by pycnodysostosis, a rare autosomal recessive skeletal dysplasia, caused by a deficiency of cathepsin K. Generally, these patients have impaired bone resorption in the presence of a normal or increased number of multinucleated but dysfunctional osteoclasts. The authors described for the first time a normalisation of CTX levels in a patient with pycnodysostosis during the lactation period. These data have been confirmed by in vitro studies using osteoclasts derived from blood monocytes during lactation. This suggests that other proteinases, such as cathepsins L and S, could compensate for the lack of cathepsin K during the lactation period in this rare bone disease.

Na et al., in the paper entitled “Aesculetin inhibits osteoclastic bone resorption through blocking ruffled border formation and lysosomal trafficking” [8], showed the effects of aesculetin, a naturally occurring compound with anti-inflammatory and antibacterial effects, on osteoclastogenesis. In particular, they found that aesculetin inhibited osteoclast activation and bone resorption through the blocking formation and exocytosis of lysosomes. In light of their data, the authors suggested for medical use aesculetin, as a potential osteoprotective agent targeting RANKL-induced osteoclastic bone resorption.

Osteoporosis is generally considered more prominent among women than men. However, in the review by Rinonapoli et al., entitled “Osteoporosis in men: a review of an underestimated bone condition” [9], the authors showed that osteoporosis and fragility fractures have high incidence in men as well; furthermore, the risk of fatal complications in men with hip fractures is higher than that in women. The authors analysed the epidemiology, aetiologies, diagnosis, and treatment of osteoporosis in men and concluded that this condition is underscreened, underdiagnosed, and undertreated.

In the review entitled “Mechanisms of bone fragility: from osteogenesis imperfecta to secondary osteoporosis” [10], El-Gazzar et al. highlighted the main disease mechanisms underlying the development of human bone fragility associated with low bone mass. They focused on different biological pathways: type I collagen processing, WNT-signalling, TGF-ß signalling, the RANKL-RANK system and the osteocyte mechanosensing pathway. Finally, they showed that the discovery of most of these pathways has led to targeted, pathway-specific treatments.

Recent research suggests that the microbiota is involved in the regulation of bone metabolism, and that its alteration may induce osteoporosis. This is the topic of the review by De Martinis et al. entitled “The osteoporosis/microbiota linkage: the role of miRNA” [11]. In particular, the authors explored the relationships occurring between microbiota and microRNAs (miRNAs), which are a set of small non-coding RNAs able to regulate gene expression. Improved comprehension of these relations could produce novel markers for the identification and monitoring of osteoporosis, as well as aid the development of new therapeutic targets.
